# Impact of G-Quadruplexes on the Regulation of Genome Integrity, DNA Damage and Repair

**DOI:** 10.3390/biom11091284

**Published:** 2021-08-27

**Authors:** Anzhela V. Pavlova, Elena A. Kubareva, Mayya V. Monakhova, Maria I. Zvereva, Nina G. Dolinnaya

**Affiliations:** 1 Department of Chemistry, Lomonosov Moscow State University, Leninskie Gory 1, 119991 Moscow, Russia; zvereva@genebee.msu.ru (M.I.Z.); dolinnaya@hotmail.com (N.G.D.); 2 Belozersky Institute of Physico-Chemical Biology, Lomonosov Moscow State University, Leninskie Gory 1, 119991 Moscow, Russia; kubareva@belozersky.msu.ru (E.A.K.); monakhovamv@gmail.com (M.V.M.)

**Keywords:** G-quadruplex, DNA repair, G-quadruplex-binding proteins, DNA damage, genomic instability

## Abstract

DNA G-quadruplexes (G4s) are known to be an integral part of the complex regulatory systems in both normal and pathological cells. At the same time, the ability of G4s to impede DNA replication plays a critical role in genome integrity. This review summarizes the results of recent studies of G4-mediated genomic and epigenomic instability, together with associated DNA damage and repair processes. Although the underlying mechanisms remain to be elucidated, it is known that, among the proteins that recognize G4 structures, many are linked to DNA repair. We analyzed the possible role of G4s in promoting double-strand DNA breaks, one of the most deleterious DNA lesions, and their repair via error-prone mechanisms. The patterns of G4 damage, with a focus on the introduction of oxidative guanine lesions, as well as their removal from G4 structures by canonical repair pathways, were also discussed together with the effects of G4s on the repair machinery. According to recent findings, there must be a delicate balance between G4-induced genome instability and G4-promoted repair processes. A broad overview of the factors that modulate the stability of G4 structures in vitro and in vivo is also provided here.

## 1. Introduction

It is well known that DNA can adopt various sequence-dependent secondary structures distinctly different from the classical Watson–Crick double helix. Among them, G4s are currently the most abundant and examined noncanonical conformations owing to their involvement in the regulation of various cellular processes. Over the past few decades, the structure of G4s, their distribution in cells and their biological functions have been extensively studied.

G4s are four-stranded nucleic acid structures that can originate either from DNA or RNA regions containing adjacent guanosine-rich runs (G-tracts). G4s arise from the stacking of two or more G-tetrads, which are a planar cyclic arrangement of four guanine bases held together by Hoogsteen hydrogen bonds and stabilized additionally by monovalent cations coordinated in or between the G-tetrads, with a decrease in the stabilization efficiency in the following order: K^+^ > Na^+^ > NH_4_^+^ >> Li^+^. Oligonucleotide loops that connect consecutive G-tracts play an important role in the overall folding and stability of G4s. Three types of loops with propeller, diagonal and lateral orientations are typical for G4s [1]. The type of loop depends on the number and nature of the loop nucleotides, as well as strand directionality and the number of G-tetrads that they traverse. Some long loops have been found to adopt well-defined, hairpin-like structures that increase the thermodynamic stability of the G4s (for review, see [2]).

The G4 structures are highly polymorphic. Under in vitro conditions, they are influenced by factors such as the number of DNA molecules involved in G4 formation, oligonucleotide concentration and sequence, especially the number of G-tracts and their length, the type and concentration of cations in solution, the length and secondary structure of the loops, crowding conditions, the presence of different cosolutes and other biomolecules [2,3,4]. G4s can adopt right-handed parallel, antiparallel and hybrid (3 + 1) topologies characterized by different orientations of the four G-tracts in the quadruplex core and *syn-* or *anti*-guanosine glycosidic bond angles, as well as left-handed G4 structures [5]. The conformational diversity of intramolecular DNA G4s is expanded due to the detected G4-forming sequences (G4 motifs) that escape the standard QuadParser algorithm (G_n_L_1-7_G_n_L_1-7_G_n_L_1-7_G_n_, where n = 3, L = A, T, G, C) [6]. G4s with long loops (up to 21 nucleotide residues) [7], bulges that arise from the incorporation of non-guanine bases in G-tracts [8,9], G4s stabilized with just two G-tetrads [10,11] and G4s with missing G, i.e., with a G-triad instead of a G-tetrad in the quadruplex core [12], were identified. The formation of intermolecular G4s, four-stranded DNAs containing other types of tetrads consisting of A, T, C residues [13] and mixed tetrads containing Watson–Crick base pairing in the quadruplex context [14], as well as higher-order parallel-stranded G4 structures [15], further increases the conformational space of these noncanonical forms.

Some improvements in sequence-based G4 motif prediction in genomes have recently been made using a radically different G4Hunter algorithm [16]. This algorithm takes into account the G-richness and G-skewness (G/C asymmetry between complementary DNA strands) of a given region. The sequences capable of forming G4s are evolutionarily conserved. They are widely represented in the genomes of all organisms, although they are predominantly enriched in eukaryotic genomes. Bioinformatic analysis showed that G4 motifs frequently cluster at key regulatory elements such as promoter regions of many oncogenes and genes involved in growth control [17,18,19], replication origins, untranslated exon regions, immunoglobulin switch sites and recombination hotspots. A high frequency of G4 motifs is also observed in telomeric DNA and micro(mini)satellite repeats, genes of ribosomal RNA and mitochondrial DNA [20]. Compared to telomeres, the G4-forming sequences found in the promoter regions are more diverse, since the varying number and length of G-tracts leads to the potential formation of multiple G4s. G4 motifs have also been well documented in all bacterial and viral genomes available in the NCBI database [21].

The evidence of G4 formation in living cells was obtained by both indirect (such as G4-associated genome instability [22,23]) and direct approaches (the use of G4-specific, fluorescently labeled antibodies and small-molecule ligands [24,25,26,27,28], in-cell NMR spectroscopy [29] or real-time visualization of DNA G4 structures in living cells with small-molecule fluorescence probes [30]). In cells, G4s appear to form maximally during the S phase [24], indicating that their number is dependent on DNA replication, a process at which the DNA strands are transiently separated at the replication fork, allowing the single strands to fold into a G4 structure.

G4 recognition by specific cellular proteins provides functional evidence of these noncanonical forms in vivo. They play critical roles in the regulation of key biological processes in eukaryotic genomes, such as telomere maintenance, the regulation of gene expression at the transcriptional level, DNA replication initiation and DNA damage and repair, as well as promoting immunoglobulin gene recombination and programming genome rearrangements. Recently, the role of G4s in embryonic development, the most controlled process in vertebrate biology, has been established [31]. Thus, it was shown that G4s in the promoters of developmental genes enhance their transcription in zebrafish embryos, probably by favoring the binding of specific transcription factors or by keeping the DNA molecule open, thereby facilitating the re-initiation of transcription. On the other hand, G4 formation causes accidental genome and epigenome instability, associated with carcinogenesis and neurological disorders [32]. Although the functions of G4s in prokaryotes and viruses are not fully elucidated, these non-B-form structures are considered important regulators of pathogenic processes because they control the expression of virulence genes [33].

In recent years, the deleterious effects of G4s on genome integrity and their potential role in regulating the DNA repair machinery have become the subject of intense research. It has been shown that, among identified G4-binding proteins, there are many G4-resolving and repair proteins, such as special helicases and proteins involved in homologous recombination and other canonical repair pathways [34,35]. Recent findings have revealed novel functions of G4 structures in various methods of epigenetic regulation. However, a detailed understanding of the G4 effect on DNA repair and the mechanisms by which DNA replication is coupled to genetic and epigenetic instability is currently lacking. In this review, we have summarized recent data on G4-mediated regulation of these key cellular processes. We have characterized the main factors that play a dominant role in the efficiency of G4 damage, mainly the introduction of oxidative guanine lesions, as well as their removal from G4 structures by various repair pathways. Since G4 formation in the genome context, their stabilization and resolution must be regulated in a complex, coordinated manner [36], a broad overview of the factors that stabilize G4 structures in vitro and in vivo has been presented.

## 2. The Factors Modulating G4 Folding and Stability In Vivo and In Vitro

**Figure 1:**
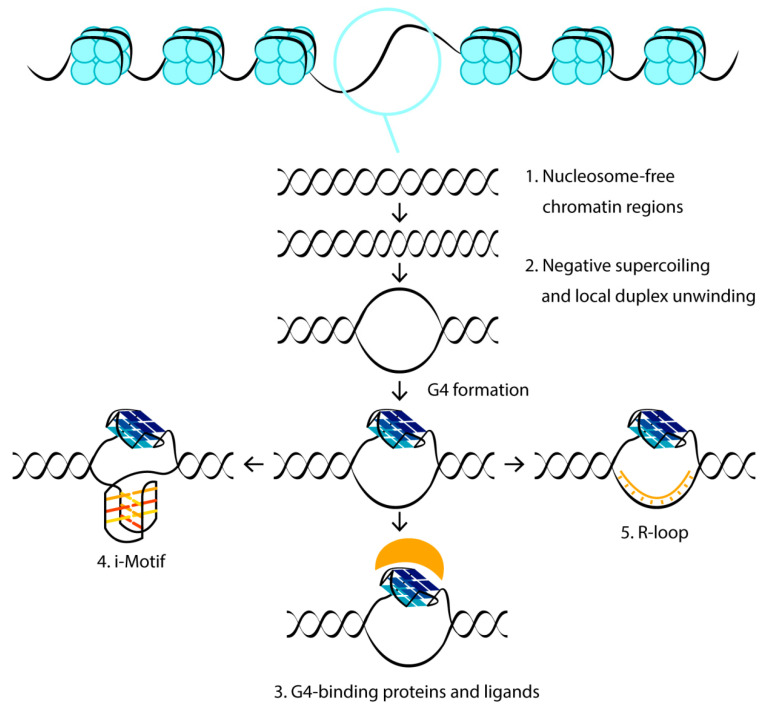
Cellular factors contributing to the folding and stabilization of G4 structures. Blue circles represent histones, G4-binding proteins and low molecular weight ligands are shown in yellow.

Depending on accepted algorithms for G4 motif searching, it was predicted that somewhere between 300,000 and 1.5 million sequences in the human genome are capable of folding into the G4 structures [16,37], and approximately 700,000 of these were detected using a G4-seq dataset [38]. An indirect G4 mapping strategy developed with the help of computational tools was presented recently by experimental small-molecule-based human genome G4 profiling [39]. However, the actual amount of G4s present at any given moment is likely to be much lower because the formation of these dynamic structures is controlled by numerous opposing factors (Figure 1). Moreover, many cellular proteins that unfold G4s dramatically suppress their formation [34].

### 2.1. Chromatin Structure

In living cells, the first constraint to G4 formation is the presence of a chromatin structure, since the interaction between DNA and histones actually helps to maintain DNA in the B form. This conclusion is supported by the high G4 density observed at DNase I-hypersensitive, nucleosome-free DNA regions [17,19]. Further analysis supports the notion that the formation of a non-B-DNA structure influences the occupancy and positioning of nucleosomes in chromatin [40]. Moreover, the G4 structures are largely enriched at the boundaries of the topologically associated domains. Since the binding sites of the architectural proteins are also abundant at the DNA domain intersections, and the protein occupancy is strongly correlated with the G4 content, adjacent DNA boundaries can frequently interact with each other, suggesting a new mechanism in which G4s regulate gene expression from afar through three-dimensional interactions [41].

### 2.2. Local Density of Negative DNA Supercoiling

With the exception of single-stranded telomeric DNA, all genomic G-rich sequences are always present along with their C-rich complements, and G4 formation competes with the maintenance of the Watson–Crick duplex. The energy for local DNA melting can probably come from negative torsional tension. In some cases, negative supercoils are generated by elongating RNA polymerase, behind which they can be transformed into strand separation, and this would allow the formation of G4s in the melted G-rich regions [42]. However, detailed analysis of G4 folding in relaxed and negatively supercoiled plasmid DNAs revealed that negative superhelicity is not sufficient to drive the formation of G4 in plasmids in vitro [40,43]. Even though the local density of negative torsional tension may accumulate at higher levels in chromatin than in a plasmid in vitro [43], these findings are in contrast with results showing the ready formation of other non-B-DNA structures (Z-DNA, H-DNA, cruciforms, etc.) in negatively supercoiled plasmids [44,45,46,47]. However, the kinetic pathway from double-stranded DNA to intramolecular G4 is likely to be very different. Firstly, G4 folding necessarily involves local melting of duplex regions with high G/C content. The kinetic barrier to G4 formation will therefore be much higher than for other alternative structures. Secondly, cruciforms and H-DNA can form via a “bubble” or “zipper” mechanism, in which only a small region of the duplex unfolds, forming a local non-B-DNA structure, which is subsequently propagated into neighboring sequences. In contrast, assembly of the first G-tetrad will require the extrusion of a relatively long single-stranded region [48,49,50]. Nevertheless, these results contrast with two more recent studies that suggested supercoil-dependent G4 formation in the *c-Myc* and *VEGF* promoters [51,52]. This contradiction in the effect of superhelicity on promotion of G4 formation can be explained by various G4 motifs investigated in [40,43], on the one hand, and in [51,52], on the other hand, as well as by different density of negative supercoiling inDNA plasmids or used ionic conditions. It seems reasonable to suppose that other processes that locally melt the double-stranded DNA in vivo, such as replication, transcription, recombination and DNA repair, might act together with DNA supercoiling to promote G4 formation.

### 2.3. G4-Recognizing Cellular Proteins and Small-Molecule Ligands

One more driving force for G4 folding in vivo may be G4 stabilization by cellular proteins such as breast cancer type 1 susceptibility protein (BRCA1), poly(ADP-ribose) polymerase 1 (PARP-1), Myc-associated zinc-finger (MAZ) protein, mutant p53 protein, proteins of shelterin complex, topoisomerase I, nucleoline and nucleophosmin [2,53]. In contrast, G4 helicases normally act to resolve quadruplex structures before replication to maintain genomic stability [34]. In vitro, low-molecular-weight ligands with different binding affinity, structural selectivity and G4-stabilizing properties [54] can be used as G4 stabilizers to shift the DNA duplex-to-G4 equilibrium. The G-tetrad faces on the top and bottom planes of G4s, as well as the quadruplex loops, are often sites for interaction with G4-binding proteins and small molecules.

### 2.4. Formation of an I-Motif on a C-Rich Complementary Strand

An important factor that can shift the folding equilibrium toward the G4 structure is the formation of a stable i-motif on the complementary strand. i-motifs are four-stranded DNA secondary structures that form in cytosine-rich sequences. Stabilized by acidic conditions, they consist of two parallel-stranded DNA duplexes held together in an antiparallel fashion by intercalated cytosine–cytosine base pairing. There is conflicting evidence as to whether the G4 and the i-motif can be folded simultaneously or whether they are mutually exclusive. On the one hand, the formation of both four-stranded structures, slightly offset from each other in the *c-Myc* promoter inserted into the supercoiled plasmid, was established using enzymatic and chemical footprinting [51]. On the other hand, it was shown using the laser tweezers-based single-molecule technique [55] that the G/C-rich insulin-linked polymorphic region forms only one four-stranded structure, but not both, under conditions that favor the formation of G4 and the i-motif—pH 5.5, 100 mM K^+^. A more general conclusion was made in the paper [56]. Although, in many G/C-rich regions, including human telomeres, the *hINS* and *hTERT* promoters, G4 and i-motif are mutually exclusive, the displacement of the sequences forming the G4 and i-motif in opposite DNA strands leads to the simultaneous formation of both structures, suggesting that the mutual exclusion between the two quadruplexes is controlled by steric hindrances. The simultaneous formation of both noncanonical structures, probably offset from each other in complementary strands, was observed using population analysis on the optical tweezers-based unfolding profiles in the *BCL-2* promoter sequence. Recently, it has been shown that multiple G-tracts present on both DNA strands of duplex Rif1-binding sequences can adopt specific higher-order structures. Their generation on a C-rich strand strongly depends on the G4 formation on a G-rich strand, and they are not likely to be the i-motif [57].

The interdependent formation of the G4 and i-motif, governed by appropriate sequences, can provide a finer coordination of the opposite signaling activities of these two alternative DNA forms in vivo [58]. In addition, they are predominately considered isolated structural targets for anticancer therapy.

Interestingly, in a recent paper [59], it was shown by immunofluorescence analysis that small-molecule-induced G4 stabilization destabilizes i-motifs, and vice versa, indicating further interplay of both structures as an important mechanism of gene regulation in human cells. This effect can probably be explained by an increase in mutual steric hindrances arising from the small-molecule ligand binding to one of the noncanonical DNA structures.

### 2.5. Exclusion of the C-Rich Strand from the DNA Duplex–G4 Equilibrium

Another factor that may help to overcome the kinetic barrier of G4 folding is the exclusion of the C-rich strand from the DNA duplex–G4 equilibrium by binding to a nucleic acid other than the original complementary DNA region.

Thus, the *BCL-2* promoter sequence inserted into the negative supercoiled DNA plasmid was able to form G4 only after the addition of short peptide nucleic acids that bind to the complementary C-rich strand region, displacing the G4 motif, thereby making it available for quadruplex formation [60].

R-loop formation is also the driving force for G4 folding. R-loops are three-stranded structures in which nascent G-rich transcript hybridizes with the DNA template strand, leaving the coding G-rich DNA single-stranded. R-loops form co-transcriptionally at sites exhibiting G/C-skew due to the increased thermodynamic stability of G-rich RNA hybridized with a C-rich DNA strand. This facilitates the possibility that G4 may arise on a displaced G-rich DNA strand at R-loops. Coupled R-loop/G4 structures, termed G-loops, have been demonstrated by electron microscopy and biochemical methods both in vitro and in *Escherichia coli* [61]. G4 arising in one strand is largely supported by an R-loop in the opposite strand, and vice versa. Accordingly, the formation of the R-loop is highly favored by the stabilization of the displaced strand by G4s and/or single-stranded DNA-binding proteins, given that the entire length of the R-loop displaced strand can exceed hundreds or thousands of nucleotides, as shown in genome mapping studies [62]. Such dynamic R-loop/G4 interplay could have significant biological implications for replication and DNA repair, as discussed in a recent review [63].

Colocalization of G4s and R-loops was visualized in cultured cells by immunofluorescence microscopy using specific antibodies BG4 and S9.6. BG4 is a useful tool to detect quadruplexes in living cells because it specifically recognizes intramolecular and intermolecular DNA and RNA G4s with high affinity (*K*_d_ = 0.5–2 nM). S9.6 is a mouse monoclonal antibody that binds to DNA/RNA hybrids with nanomolar affinity [64]. Monitoring of co-transcriptional G4 formation by single-molecule fluorescence resonance energy transfer (FRET) showed that R-loop formation precedes and facilitates G4 assembly. Moreover, G4s remain stable for a long time, even after disruption of R-loops—for example, during their treatment with RNase H. G4s can accumulate as a result of multiple transcription, facilitating, in turn, R-loop formation in subsequent rounds of transcription and, thus, providing positive feedback between G4s and R-loops [65].

A radical way to exclude the C-rich region opposite to the G4 motif embedded into the DNA duplex has recently been proposed for in vitro models. For this, partly complementary strands were hybridized, one of which contained the G4 motif flanked with oligonucleotide fragments, while the opposite strand lacked a site complementary to the G4-forming insert. Therefore, unlike the surrounding sequences, the central moiety cannot be converted to B-DNA and compete with the G4 formation. Steric factors, determined by quadruplex topology, strongly influence the ability of G4 to coexist with a neighboring duplex in the same DNA structure. Parallel intramolecular G4s, usually formed in promotor regions, have 5′- and 3′-ends on opposite sides of the G4 core, thereby facilitating its placement inside the double helix without any structured linker [35]. In contrast, antiparallel G4s enchain ends on the same side, which requires a duplex linker to accommodate the G4 structure in B-DNA [66]. Another approach involves the use of a random sequence instead of a sequence complementary to the G4 motif [67].

## 3. Genetic Instability during DNA Replication through G4-Containing Regions and Error-Prone Repair of G4-Induced DNA Double-Strand Breaks

In addition to regulatory functions, G4 formation can promote genome instability, implicating G4s in disease and evolution. These noncanonical DNA structures constitute an obstacle to replication machinery. G4s can interfere with the replication of both the lagging and leading strands [34]. The defects in the systems that contribute to G4 resolution can stall a replication fork, thereby giving rise to DNA double-strand breaks (DSBs)—one of the most deleterious DNA lesions, which can promote mutagenesis (e.g., inversions, recombination, mutations and deletions), loss of genetic information and deregulation of the genome. These aberrations are significant drivers of human diseases such as cancers and genetic disorders. DNA damage arising due to G4 formation can be transferred through several mitotic divisions to daughter cells. During the first round of DNA synthesis, a single-strand break occurs, which leads to DSBs in the next generation of daughter cells in the second round of replication [68]. It is well known that G4s interfere with the function of DNA polymerases in vitro. Failure to bypass the G4 barrier has been demonstrated for various human polymerases involved in both DNA replication and repair [69]. A quantitative analysis of the correlation between the replication rate by the Klenow fragment of DNA polymerase, on the one hand, and the G4 topology and thermal stability, on the other hand, revealed that hybrid telomeric G4 was a less effective inhibitor of DNA polymerase processivity than antiparallel or parallel G4 structures [70]. It turned out that only those G4s that have very short loops (less than 4 nucleotides in length) and, accordingly, high thermal stability, cause replication-dependent genome instability [71]. Evidence of the negative G4 impact on DNA polymerization in vivo was obtained by analyzing the cellular action of small-molecule G4 ligands, which stabilize these structures. For example, local epigenetic G4-dependent transcriptional reprogramming induced by G4 ligands has been described [72]. Treatment of immortalized human fibroblasts with the ligand RHPS4 resulted in DNA lesions detected by foci of phosphorylated histone gammaH2AX, a DSB biomarker, exclusively in S-phase cells. This ligand particularly affected the replication of the G-rich lagging strand of the telomeres and resulted in telomere aberrations known as the “fragile telomere” phenotype [73]. Genome-wide analysis of DSB points associated with somatic copy number alterations from 2792 cancer samples showed significant enrichment for G4-prone sequences, which are characterized by abnormal hypomethylation, in the vicinity of DNA breakpoint hotspots [74]. The authors proposed that hypomethylation in genomic regions enriched with G4s acts as a mutagenic factor driving the tissue-specific mutational landscape in malignant cells. It has been shown that changes in methylation are heritable in successive cell division [72].

**Figure 2:**
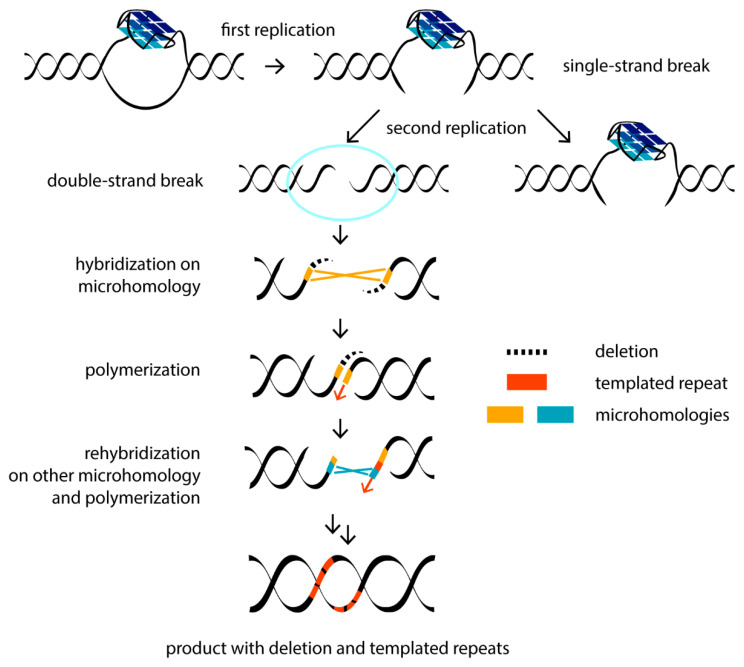
Scheme of G4-mediated genesis of DSBs after the second round of replication and subsequent DSB repair by the TMEJ mechanism with the formation of typical mutational patterns. Hybridization based on microhomology (in orange) and DNA synthesis by DNA Pol θ results in the deletion of sites external to microhomology (dashed line). Switching the template to another microhomologous sequence (in blue) leads to a Pol θ-mediated sequence insertion adjacent to the microhomology (in red). Several template switches can result in more reinsertions.

The nature of DNA mutations promoted by G4-mediated replication stalling has been extensively studied in the engineered genome of the worm *Caenothabditis elegans* [75]. Using a created selectable system, a noncanonical DNA break repair mechanism was identified. The DSBs are mainly repaired by two pathways: non-homologous end joining (NHEJ) and homologous recombination (HR). Each of them brings a certain risk of mutagenesis. However, an atypical mutation profile in *C. elegans* (extremely narrow size distribution of mutations, the occasional presence of templated insertions, etc.) justified an alternative DSB repair pathway, which requires polymerase Theta (Pol θ), the absence of which leads to the profound loss of sequences surrounding G4 motifs. The Pol θ-mediated end-joining (TMEJ) pathway has been found to repair the replication-associated DNA breaks that are excluded from repair through HR [68]. Due to its mechanism of action, TMEJ is also intrinsically mutagenic and operates at the end of the S phase of the cell cycle. Pol θ uses microhomology (1–2 nucleotide overlap at the two-ended DSBs) as a primer to synthesize short 1–25-nucleotide stretches to fill the gap. This may be followed by repeated hybridization at another microhomologous site and re-synthesis (Figure 2). For this reason, along with genomic deletions at the repair site, Pol θ leaves behind the recognizable “scars”—repeated insertions surrounding the deleted site [76]. Using the *C. elegans* model, it was found that G4 DNA can be conserved through multiple mitotic divisions, resulting in DSBs and the deletion of around 120 base pairs following the TMEJ pathway [77].

The HR repair involves templated DNA synthesis using an intact homologous DNA sequence. However, if an unresolved G4 structure is formed in the sister chromatid, the HR pathway is not effective [78]. At the same time, HR is able to prevent G4-mediated replication fork collapse, which precedes DSB generation, by reinitiating replication and switching to a different substrate in the region of G4 formation. Direct evidence was obtained in vivo for the involvement of human PIF1 helicase in the HR repair of G4-containing DNA through interaction with the HR-associated protein BRCA1 [79].

## 4. G4-Based Somatic Hypermutations in Immunoglobulin Genes Induced by Activation-Induced Cytidine Deaminase

In addition to the generation of DSBs promoted by G4-mediated replication stalling, G4s are involved in genome destabilization through other mechanisms. To enhance the specificity and functionality of antibodies, mammalian immunoglobulin genes in B cells undergo two DNA alteration events that are triggered by activation-induced cytidine deaminase (AID): somatic hypermutation and class switch recombination. Particularly good AID substrates in vitro are G4-forming DNAs, the sequences of which mimic the mammalian immunoglobulin switch region and contain tandem G-tracts interspersed with recombination hotspots: 5′-AGCT-3′ [80]. It was suggested that the G4 structures are formed co-transcriptionally on the G-rich nontemplate strand and contribute to the R-loop stability. Two types of G4s were used as substrates for deamination; intermolecular parallel G4, formed by one G-tract containing flanking sequences, and mixed inter- and intramolecular G4. It was found that AID deaminase does not recognize the G4 core itself, but instead simultaneously interacts with two adjacent DNA loops and uses cytidine residues located three nucleotides from the G4 core as preferred sites for deamination. Moreover, G4 substrates induce cooperative AID oligomerization, which can promote cluster mutations in immunoglobulin regions [81].

## 5. DNA Helicases Unfold G4 Structures to Prevent G4-Mediated Genetic and Epigenetic Instability

To mitigate the deleterious effects of endogenous DNA G4s, many special cellular mechanisms have been developed that facilitate replication across structured DNA regions. They include the removal of G4s by helicases or nucleases that can specifically bind and unwind or cleave these noncanonical structures. Helicases are molecular motors that use the energy of ATP to locally unwind double-stranded DNA. This stage is required for virtually all key cellular processes, including replication, transcription, repair, recombination and chromosome segregation. DNA helicases can destabilize certain G4 structures in vitro in an ATP hydrolysis-dependent manner [82]. Only a limited number of DNA helicases are capable of unwinding G4 structures. Eukaryotic cells have at least ten helicases that have been shown to play a role in G4 metabolism in vitro: DNA2 and PIF1, which are members of superfamily 1; RecQ helicases BLM and WRN, belonging to the superfamily 2; the superfamily 2 Fe-S helicases FANCJ, DDX11, RTEL1 and XPD; and DEAH box helicases DHX9 and DHX36 [34]. The directionality of G4 unwinding distinguishes the action of G4-resolving helicases. Thus, RecQ helicases require a 5′-single-stranded DNA tail in the G4 substrate (5′→3′ helicases), whereas a 3′-DNA tail is required in order for BLM and WRN to function (5′→3′ helicases). When activated in the process of G4 resolution, helicases more readily unwind double-stranded DNA during replication [83]. It is important to note that all of these enzymes play important roles in other cellular pathways. Nevertheless, to date, no DNA helicase has been identified that is solely dedicated to G4 unwinding. However, G4-resolvase 1 (also known as DHX36 or RHAU) exhibits a strong preference for unfolding G4 structures in an ATP-dependent manner but fails to unwind duplex DNA [84]. Some helicases have been shown to be involved in G4 processing in vivo. In particular, studies of the PIF1 DNA helicase family, which is conserved from bacteria to humans, revealed the colocalization of PIF1-binding sites and G4-forming sequences in the *Saccharomyces cerevisiae* genome, as shown by chromatin immunoprecipitation (ChIP). In addition, the DNA of PIF1-deficient cells turned out to be more susceptible to DSBs [85].

The mechanism and kinetics of some G4-unwinding helicases were discussed in [83,86,87]. Unlike the highly processive PIF1, which remains bound to its DNA substrate, G4-resolvase 1 is nonprocessive, since it leaves the substrate after each act of ATP hydrolysis.

Several helicases have variable functional activities and show a clear preference for binding to G4s of different topologies. Thus, DHX36 prefers parallel G4s, ATP-dependent 3′→5′ BLM equally recognizes both parallel and non-parallel G4s, and ATP-dependent 3′→5′ WRN selectively unwinds telomeric G4s [34,88]. Moreover, BLM-induced unwinding of intramolecular G4s oссurs via different mechanisms depending on the G4 surrounding (single- or double-stranded), as shown by FRET [89]. Despite differences in substrate specificity, RHAU, BLM and WRN share the same mechanism, which involves repetitive cycles of G4 unfolding leading to efficient hybridization of the G4 motif with the complementary DNA region, as demonstrated by single-molecule imaging [88].

Recent studies of *Xenopus laevis* egg extracts have established a novel genome maintenance pathway that promotes accurate G4 replication, thereby preventing genome instability. It includes a three-step G4 unwinding mechanism associated with DNA replication [67]: (1) the replicative CMG polymerase stalling near the G4 structure on the leading strand; (2) DHX36 helicase-mediated CMG bypass of the folded G4 structure, allowing the polymerase to approach G4; (3) G4 structure unwinding by the FANCJ helicase, ensuring the replication of the G4 motif. It is noteworthy that the G4 on a lagging strand does not stall the CMG, but still requires DHX36 and FANCJ to unwind. Both these G4-resolving helicases play partly redundant and overlapping roles. FANCJ, being a 5′→3′ helicase, can also create single-stranded DNA regions behind the intact G4 structure, facilitating CMG translocation. DHX36 is directly involved in G4 unwinding. This confers robustness to this pathway. In [67], DNA plasmids with a G4 motif located either in the “upper” or in the “lower” strand were used as the model systems; moreover, to stabilize the G4 structure in a duplex context, the sequence opposite to the G4 motif was noncomplementary to it.

**Figure 3:**
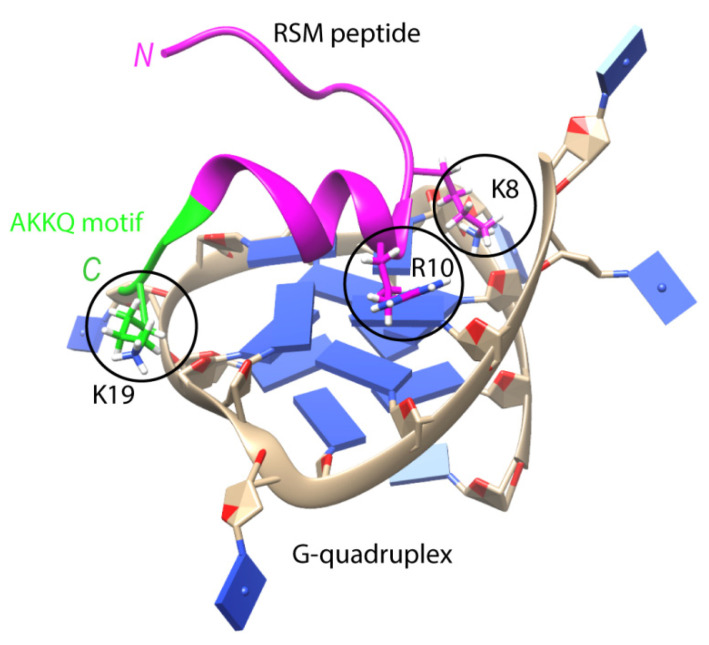
The structure of the complex between part of the DHX36 helicase (RSM peptide, in pink and green) and parallel G4 with heterocyclic bases marked in blue (PDB 2N21 [90]). Three positively charged amino acid residues (K8, R10 and K19, circled) form electrostatic interactions with phosphate groups of G4. The amino acids of the α-helix stack with the external G-tetrad, while the AKKQ motif (in green) anchors the peptide in the G4 groove.

Multifunctional helicase DHX36 (DEAN-box helicase RHAU) recognizes the parallel G4 structure using an 18-amino-acid domain at the N-terminus of the protein (RSM motif), which folds into an L-shaped α-helix with an AKKQ loop [90]. These findings are generally supported by the co-crystal structure of bovine DHX36 bound to a DNA with G4. In addition, single-molecule FRET analysis showed that G4 binding induces rearrangements of the helicase core, causing G4 to unfold one residue at a time [91]. The RSM motif covers the outer G-tetrad and clamps G4 using three-anchor-point electrostatic interactions between three positively charged amino acids and negatively charged phosphate groups [90] (Figure 3). Interestingly, this binding mode is strikingly similar to that of most small-molecule ligands selected to specifically target G4s. DHX36 has a very high affinity for G4s, with a *K*_d_ < 10 pM [92]. It has recently been demonstrated that, in addition to the previously identified specific G4-recognizing RSM motif, there is another G4-binding domain that stabilizes the G4 structure in the absence of ATP [93]. It is noteworthy that the affinity of FANCJ for G4 is approximately 100 times lower than that of DHX36 for the same substrate [94].

Helicase FANCJ is required for normal S-phase progression, indicating that it removes structural barriers to replication. This helicase also implicates the Y-family polymerase REV1 to promote the replication of G4-containing templates. REV1 is known to coordinate other polymerases—for example Pol ζ (REV3/REV7)—in a DNA repair process known as translesion synthesis [95]. REV1 is a highly specialized polymerase that incorporates only dC in DNA, and the enzyme itself dictates the identity of the incoming nucleotide. Loss of proteins known to process G4s, including FANCJ, BLM and WRN, or loss of the translesion REV1 polymerase, leads to the disruption of histone recycling, loss of chromatin marks near G4s and failure in the epigenetic regulation of gene transcription [96].

Monitoring of epigenetic instability at the single cell level provided evidence that DNA helicases are able to cooperate and function in parallel [97]. It was suggested that helicases operating in the opposite polarity (5′→3′ and 3′→5′) can work together, unwinding G4 from both ends, and a 5′→3′ helicase can collaborate with specialized polymerases, such as REV1 acting from the stalled 3′-primer terminus [97]. In addition, helicases may act sequentially with the first enzyme, remodeling G4 into a form that is more amenable to unwinding by the second enzyme [34].

Another proposed mechanism for replisome bypassing through DNA regions with G4-forming potential includes helicase DDX11 and replisome components of the fork protection complex (FPC) [98]. The Timeless protein from the FPC complex interacts with G4 and recruits the DDX11 protein with in vitro 5′→3′ G4 helicase activity, which is increased in the presence of Timeless.

Helicases WRN and BLM play an important role in telomere replication. For effective G4 processing in the S phase of the cell cycle, preliminary dissociation of the sheltering complex and telomere-bound proteins must occur. Using antibody-mediated visualization, it has been shown that the G4 folding/unwinding processes in *Stylonichia*, telomeres depend on the expression of telomere-end-binding proteins (TEBPs) [99]. Phosphorylation of TEBPβ (homolog of human TPP1 protein in ciliates) by Cdk2 kinase during the S phase prevents TEBPβ from binding to TEBPα (homolog of human POT1 in ciliates), which leads to degradation of the telomere cap protein complex. RecQ helicases (WRN or BLM in humans) associated with telomerase then implement G4 unwinding [100]. The increased G4-binding affinity of these helicases is mediated by the conserved RecQ C-terminal domain.

Stabilization of telomeric G4s with small-molecule ligands inhibits the unwinding activities of FANCJ and several RecQ helicases, including WRN and BLM, during telomere replication, resulting in DNA replication stress, accelerated telomere shortening, chromosomal aberrations including chromosome fusion and cell apoptosis [101]. Both BLM and the other 5′→3′ DNA helicase RTEL1 are required to repress the fragile telomere phenotype [73,102].

Certain DNA helicases are genetically linked to human diseases characterized by chromosomal instability. Thus, Werner’s syndrome and Bloom’s syndrome are characterized by autosomal recessive mutations in the genes encoding the DNA helicases WRN and BLM, respectively [103,104]. While Werner’s syndrome leads to premature aging (progeria) and a predisposition to age-related diseases, Bloom’s syndrome is associated with increased sister chromatid exchange and a very high incidence of various types of cancers. Another helicase, FANCJ, is associated with a genetic disorder called Fanconi anemia. FANCJ-depleted human cells from patients with Fanconi anemia accumulate large 50–300-nucleotide deletions in the genome, with typical TMEJ insertions in the immediate vicinity of G4-forming regions [105].

## 6. Hydrolysis of G4 Structures by Deoxyribonucleases as a Way to Maintain Genome Integrity

Although G4s, due to their stable compact secondary structure, are resistant to nuclease degradation, a multifunctional enzyme DNA2, which is critical for maintaining telomere integrity, can bind and unwind G4s in an ATP-dependent manner in vitro [106] and cleave them [107]. Yeast DNA2 exhibits 25-times higher affinity for G4 formed by telomeric repeats than for single-stranded DNA of the same sequence. Human DNA2 is also involved in the nucleolytic processing of DNA containing G4s. Interestingly, single-stranded DNA is not a competitive inhibitor of G4–DNA2 binding, which suggests different modes of interaction between DNA2 and G4, on the one hand, and DNA2 and single-stranded DNA, on the other. Compared to single-stranded DNA, the efficiency of DNA2-mediated G4 cleavage decreases in a G4 structure-dependent manner. However, the interaction of DNA2 with replication protein A (RPA) elevates nuclease-induced DNA cleavage at the 5′-end of the G4 core. DNA2 deficiency in mouse cells leads to telomere replication defects, increased levels of fragile telomeres and chromosome segregation errors. Such telomere defects are enhanced by low molecular weight G4-stabilizing ligands such as macrocyclic TMPyP4 and telomestatin [108]. These findings suggest that DNA2 operates during telomere replication to mediate the resolution of telomeric G4s. Moreover, DNA2-mediated G4 cleavage is likely to maintain the stability of other genome regions during the S phase of the cell cycle.

In addition to DNA2, a number of other nucleases exhibit G4-dependent DNA binding and cleavage activities—for example, the product of the *S. cerevisiae KEM1* gene [109], as well as human Flap endonuclease 1 (FEN1) [110] and exonuclease 1 (EXO1) [111]. FEN1 is involved in lagging-strand DNA replication, base excision repair (BER) and HR, and it contributes to the stability of telomeres, ensuring their efficient replication. Exonuclease EXO1 plays a key role in G4 resolution and replication through telomeric G4s. When the replication fork encounters the G4 structure, EXO1 cleaves nascent DNA proximal to G4 in a 5′→3′ direction to facilitate DNA replication. EXO1 deficiency provokes replication fork collapse and DSB generation; the subsequent aberrant repair leads to increased genome instability, which is manifested in telomere shortening.

## 7. Guanine Oxidation Alters the Folding of G4s and Reduces Their Thermal Stability

**Figure 4:**
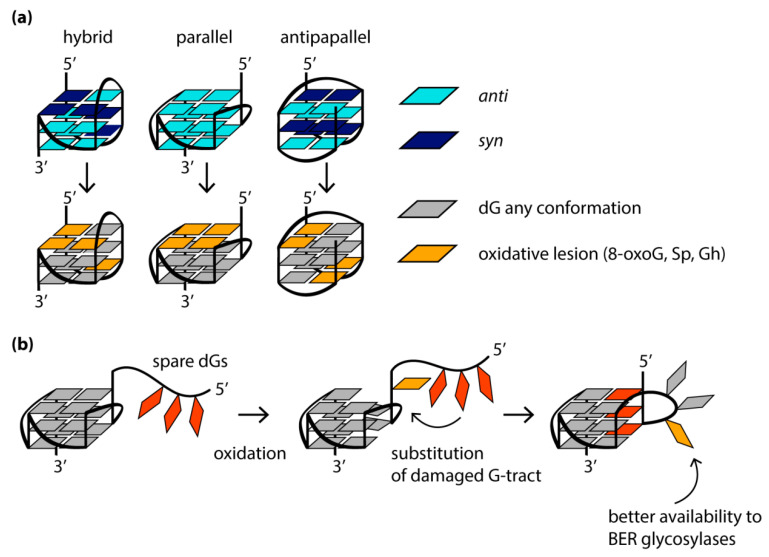
(**a**) Positions in G4s of different topologies that are most susceptible to oxidative damage. (**b**) Replacement of damaged guanine in the G4 core with available dG in loops or spare G-tracts helps to maintain the G4 structure and increases the accessibility of damaged dG residues for repair proteins.

G4 formation can lead to replication fork stalling, accumulation of deletions/mutations, genomic copy number alterations and a high recombination frequency. As shown in model organisms (*C. elegans* and *S. cerevisiae),* as well as in human tissue culture, changes in the regulation of the G4 structure lead to genome instability. G4s represent a strong block for replication and transcription, not only by themselves but also due to oxidative lesions, to which DNA regions containing a large number of guanine bases are especially susceptible. It is well-known that DNA oxidation occurs mainly at guanines, since they have the lowest redox potential among all native nucleobases [112]. One of the most common and well-documented oxidative guanine lesions is 8-oxo-guanine (8-oxoG) [113]. Molecular dynamics simulation and CD spectroscopy studies showed that the single guanine-to-8-oxoG substitutions within the quadruplex-forming sequence alter the folding of G4s in a location-dependent manner [114,115], significantly destabilize G4 structure and even lead to its unfolding due to a loss of a Hoogsteen hydrogen bonds within a G-tetrad and/or a steric clash between the proton H7 of 8-oxoG and the amino group of the neighboring guanine [116,117]. The oxidation of those guanosine residues that adopt the *syn*-conformation in the DNA G4 structure is accompanied by only minor structural changes and, as a consequence, poor G4 destabilization, while the oxidative modification of guanosines in the *anti*-conformation leads to significant conformational perturbations. Since 8-oxoG derivative of dG is known to favor a *syn*-conformation due to the bulky substituent at the 8 position of the guanine base, there is a fundamental difference in the behavior of 8-oxoG-containing parallel G4s with all dGs in *anti*-conformation and antiparallel G4s. In the latter case, the mutual orientation of the strands correlates with the *syn-* or *anti*-conformations of dG residues, changing both inside the G-tetrad cycle and along the G4 axis. For this reason, some modified DNA oligonucleotides with human telomeric repeats, which contain 8-oxoG lesions at the positions for adopting the *syn*-conformation in the original antiparallel or hybrid (3 + 1) G4 structures, are able to fold into G4s, which are stable under physiological conditions. In contrast, in parallel quadruplexes formed in promoter regions, all dGs exhibit a preference for *anti*-conformation, so that a single 8-oxoG substitution can severely disrupt the structure and stability of such G4s (Figure 4a).

To mitigate this effect, the oxidative damage of the guanine bases localized in certain positions of the G4 motif can cause rearrangement of the G4 structure, ensuring its preservation. Thus, the 8-oxoG and apurinic/apyrimidine (abasic) sites (processed products of 8-oxoG derivatives of dGs) in G4 can recruit the guanosine residues from G4 loops (Figure 4b). If there are no “spare” dGs in the sequence that can replace the damaged one, destabilization of a separate G-tract or exclusion of certain guanosines from the G4 structure is possible to preserve the partially formed quadruplex. Complete G4 unfolding usually correlates with destabilization of the central G-tetrad and the concomitant release of both central cations [114].

It is known that 8-oxoG is susceptible to further oxidation to guanidinohydantoin (Gh) and two spiroiminodihydantoin (Sp) diastereomers. The action of free radicals on guanine residues involved in G4 formation, among which the most susceptible to oxidation are guanines in the G4 loops and those adjacent to the 5′-end of the G4 core (Figure 4a), mainly leads to the generation of Sp [118]. The amount of Sp among the guanine oxidation products in the G4 context decreases when the reaction is carried out under reducing conditions similar to those observed in vivo. Similar to 8-oxoG, Gh and the Sp are not capable of Hoogsteen H-bonding in the G-tetrads; moreover, they are not planar and, therefore, cannot π-stack in the G4 core. The introduction of any oxidative guanine lesions was shown to cause the human telomeric G4s to adopt substantially different topologies, including even a parallel one, with a significant reduction in G4 thermal stability, and these effects are highly position-dependent.

## 8. Repair of Oxidative Base Lesions Located in Various Positions of the G4s by BER Enzymes

### 8.1. Removal of Oxidative Guanine and Thymine Lesions from DNA G4s

**Figure 5:**
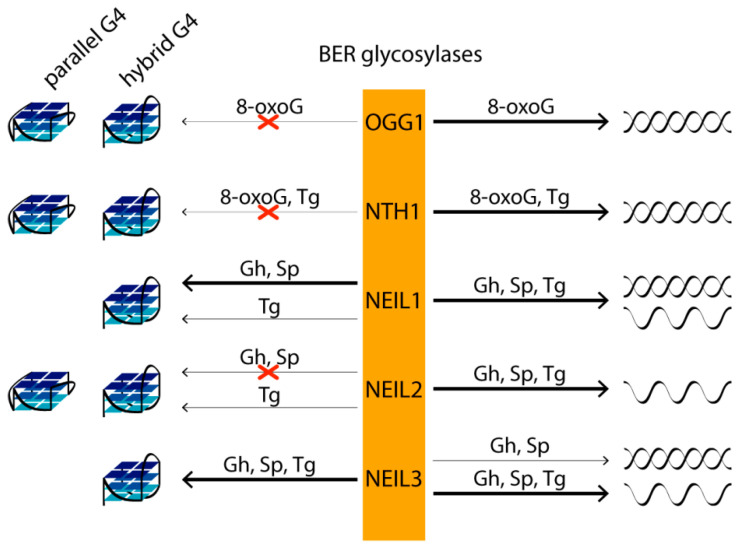
The relative preference of BER glycosylases toward oxidative guanine lesions in different structural contexts (various G4 topologies (left) versus single- and double-stranded DNAs (right)) as substrates for repair. The type of damage is shown above the arrows, the thickness of which reflects the repair efficiency: the thicker lines represent the better substrate, the thinner lines designate the relatively weaker enzyme activation, while the crossed arrows indicate the lack of the corresponding BER glycosylase activity against a specific oxidative lesion in a definite context.

If the lesion is not repaired prior to its encounter with the replication fork, there are two possible scenarios. In the first, the base lesion blocks DNA polymerase and is thus potentially lethal. In the second, it is bypassed by the polymerase and may be mutagenic depending on its ability to mispair. Moreover, 8-OxoG may mispair with adenine, causing the dG to T transversion mutations, while Gh and Sp, which are capable of mispairing adenine and guanine, can also efficiently block DNA polymerase as a product of thymine oxidative damage, thymine glycol (Tg). In human cells, five DNA glycosylases from the BER pathway—OGG1, NTH1, NEIL1, NEIL2 and NEIL3—repair various oxidative DNA base lesions. The first step of BER involves the detection of the aberrant base by highly specific DNA glycosylases. Then, one of the enzymes removes the lesion from the DNA backbone, leaving an abasic site, which is further processed and repaired by AP endonuclease, DNA polymerase and DNA ligase through a short-patch or long-patch BER pathway. The main targets of OGG1 and NTH1 are, respectively, oxidized purines and pyrimidines in double-stranded DNA. DNA glycosylases NEIL have broader substrate specificity. NEIL1, which mediates the pre-replicative repair of oxidized bases in the human genome, removing lesions before they are encountered by the replicative DNA polymerases, excises pyrimidine lesions—in particular, Tg and 5-hydroxyuracil—from double- and single-stranded DNAs. Pyrimidine and some purine lesions in single-stranded DNA are recognized by DNA glycosylases NEIL2 (transcription-coupled repair) and NEIL3. Although 8-oxoG is not a substrate for NEIL DNA glycosylases, its further oxidation products, Gh and Sp, are the best targets for all three enzymes [119] (Figure 5).

In vitro studies of G4s’ impact on oxidative DNA damage repair have shown that 8-oxoG, Gh and Sp lesions are not removed by OGG1, NEIL1, NEIL2, NEIL3 and NTH1 from the parallel-stranded G4s formed at biologically relevant K^+^ concentrations and derived from *VEGF* and *c*-*Myc* promoter sequences [119]. However, Gh and Sp located in the central G-tetrad and at the 3′- but not at the 5′-end of the G-tract in hybrid/antiparallel telomeric G4s are effective substrates for NEIL1 and NEIL3 [119]. At the same time, the removal of Gh and Sp from the DNA duplex by the same DNA glycosylases is much less position-dependent compared to that for G4 structures. Gh and Sp in all studied positions of the DNA duplex are better substrates for NEIL1 than similar lesions in telomeric G4. NEIL3, on the other hand, shows a preference for lesions in the G4 context and is more active against damaged G4 than against a corresponding DNA duplex with the same G-rich strand sequence [119]. Since the absence of NEIL3 results in a fragile telomere phenotype, repairing base damage in telomeres may be important for proper replication of telomere regions and for maintaining their integrity. It should be noted that the conservative RG-rich motif revealed during the analysis of the amino acid sequences of all known human G4-binding proteins was also identified in the NEIL1 and NEIL3 glycosylases [120]. As for NEIL2, this human glycosylase did not remove Gh from DNA G4, despite its efficient recognition of the same damage in single-stranded DNA [119] (Figure 5).

Although none of the DNA glycosylases initiated 8-oxoG repair in any of the G-tetrads of Na^+^-coordinated telomeric G4 with antiparallel folding [121] and hybrid/antiparallel G4 formed in a K^+^ solution [119,122], the prokaryotic formamidopyrimidine-DNA glycosylase Fpg has been shown to cleave 8-oxoG in the G4 context [122]. However, the efficiency of 8-oxoG removal from the G4 structure by Fpg was reduced by more than 40% compared to that from the DNA duplex. The affinity of DNA glycosylases Fpg, OGG1, Nei, NEIL1 and NTH1 to G4 with oxidative base lesions differed only slightly from the affinity for a damaged DNA duplex. Thus, the formation of primary complexes is insufficient to activate catalytic activity in the case of damaged G4s. It is known that, during the recognition of 8-oxoG in the DNA duplex, conformational changes occur both in DNA and in the enzyme (Fpg and OGG1), including local destabilization of DNA around the oxidative base lesion, DNA bending and 8-oxoG flipping out of the double helix to the active center of the enzyme, the conformational changes of which ultimately lead to the activation of catalytic functions. A transient kinetic analysis of conformational changes in 8-oxoG-cotaining G4 upon interaction with Fpg showed the presence of more complex molecular events than for duplex substrates. In the case of OGG1, no conformational changes in the damaged G4 were observed [122].

The data on 8-oxoG repair have also been compared with those from ongoing studies of the structure and repair of DNA G4s containing other oxidized bases, particularly Tg [121,122]. Among mammalian glycosylases, mNeil3 (homolog of NEIL3 from *Mus musculus*) exhibits the greatest excisional activity towards Tg located in the loops of telomeric G4. NTH1 did not remove Tg from G4, although Tg in duplex DNA was efficiently cleaved by this enzyme. NEIL1 and NEIL2 also showed very weak glycosylase activity on the Tg-containing G4 substrate. Removal of Tg from G4 with the formation of β- and β, δ-elimination products has been demonstrated for prokaryotic endonuclease VIII (Nei) [122].

DNA glycosylases, which excise the damaged bases, provide substrates, the abasic sites, for the next step in the BER pathway. Abasic sites are one of the most abundant DNA lesions in genomes and their repair is essential for cell survival. To measure human APE1 endonuclease activity on hybrid DNA G4s, oligodeoxyribonucleotides containing tetrahydrofuran (a stable abasic site analog) are commonly used. The presence of a tetrahydrofuran residue at various positions of G4 led to a 20 °C drop in quadruplex melting temperature values compared to nondamaged G4. An abasic site located in G4 DNA was shown to be hydrolyzed by APE1 200-times slower than in double-stranded DNA. At the same time, the binding efficiency of the enzyme to both DNAs containing the abasic site did not differ [123]. It is known that the APE1 interacts with G4 through lysine residues (K27/31/32/35), which are present in the unstructured N-terminal region of the protein [124]. Acetylation of these lysine residues leads to a loss of positive charge, which reduces the endonuclease activity of APE1. The available crystal structures of APE1 complexes with DNA duplexes containing the abasic site show that the enzyme recognizes the lesion via contact not only with the abasic-site-containing DNA strand through interaction with several nucleotides flanking the damage, but also with the complementary DNA strand. In addition, significant DNA bending is required for the abasic site to be located in a special cavity of the enzyme prior to the catalysis. These structural requirements are not met in the case of G4 structures, which probably explains the low activity of APE1 towards G4 compared to B-DNA. Despite its low efficiency, APE1 hydrolyzes the G4 substrate containing the tetrahydrofuran residue both in the G4 loop and in the quadruplex core; in the latter case, the catalytic activity of APE1 is five-times higher [125]. 

According to a pre-stationary kinetics study of the APE1-mediated cleavage reaction of tetrahydrofuran-containing G4, the catalytically active complex formation after the primary binding of APE1 to G4 is the rate-limiting step of the process. Other studies [119,124] showed the dependence of the efficiency of abasic site cleavage by endonuclease APE1 on the quadruplex topology: the enzymatic activity of APE1 on telomeric G4, which adopts a parallel conformation, was significantly higher than that for hybrid G4. Antiparallel telomeric G4s with a tetrahydrofuran site are also cleaved by APE1 more efficiently than hybrid ones. Thus, it was concluded that APE1 cleaves tetrahydrofuran in Na^+^-coordinated telomeric antiparallel (basket) G4 DNA but not in K^+^-coordinated hybrid G4. In addition to the endonuclease cleavage of G4 at the tetrahydrofuran site, the interaction of APE1 with both damaged and intact G4s leads to slow 3′-5′-exonuclease degradation of DNA.

In general, oxidatively damaged guanines in the context of the G4 represent poorer substrates for BER compared to duplex DNA/single-stranded DNA, with only a few examples of elevated or comparable efficiency of recognition and removal of specific lesions at certain G4 positions by particular BER glycosylases.

### 8.2. An Extra G-Tract in the G4 Motif Allows Alternative Quadruplex Folding and Facilitates the Repair of Oxidative Base Damage by the NEIL Glycosylases

NEIL glycosylases, including NEIL2, remove Gh from telomeric G4 formed by five repeats of 5′-TTAGGG-3′ much more efficiently than from the commonly studied four-repeat G4 DNA [119]. It has been hypothesized that an extra G-tract induces alternate G4 folding with the lesion-containing repeat extruded from the G4 core. As a result, lesions in the 5′- or 3′-terminal repeats become part of the 5′- or 3′-tails of the hybrid G4, respectively, and when the lesion is in the middle repeat, the latter is a part of an extended edgewise loop. Thus, an alternative folding mechanism makes oxidized guanines more accessible to NEIL glycosylases (Figure 4b). Consequently, the fifth G-tract in the *VEGF* gene promoter, acting as a reserve in the event of damage to one of the main four G-tracts, contributes to the extrusion of the damaged G-tract from the G4 core, thereby making it available for repair by DNA glycosylases NEIL1, NEIL2 and NEIL3 [118].

Thus, the plasticity of DNA G4 structures can facilitate the repair of the emerging oxidative base lesions in regulatory G4s, reducing genome instability in these regions. It should be noted that the promoters of many oncogenes, including *c-Myc*, *HIF-1α*, *KRAS*, *Bcl*-2, *RET*, *HSP90*, *PDGF* and *AR*, contain an additional fifth G-tract in close proximity to the G4 motif. For example, the promoter region of the *c-Myc* gene with five G-tracts forms two alternative G4 structures, in which two different sets of four G-tracts are involved [126]. In this case, the appearance of an AP site or 8-oxoG in one of the G-tracts shifts the equilibrium towards the G4 structure, where the intact G-tract is involved in the G4 core.

### 8.3. Effect of 8-oxoG in G4 Motif on BER-Mediated Regulation of Transcription

According to recent studies, oxidative guanine lesions in the G4 context may act as epigenetic regulators in transcription processes. Sensitive to oxidative stress, they may be important for the expression regulation of oncoproteins, as well as proteins involved in the elimination of oxidative stress consequences (for example, NEIL3 [127], NTHL1, PCNA and RAD17). To a greater extent, this mechanism of transcription regulation has been studied for the *VEGF* oncogene promoter. It is known that cancer cells are characterized by high metabolic rates, usually associated with an increased level of reactive oxygen species. For this reason, the basal level of 8-oxoG in cancer cells is up to 12-fold higher than in normal cells [128,129]. It was shown that 8-oxoG in the G4 motif of the *VEGF* promoter in the noncoding strand increases the expression of the reporter gene under the control of this promoter through a mechanism associated with the BER pathway [130]. The BER proteins OGG1 and APE1 were found to be required for the activation of gene transcription. The ability of the 8-oxoG to influence folding of the G-rich promoter sequence into the G4 structures was also an important factor.

**Figure 6:**
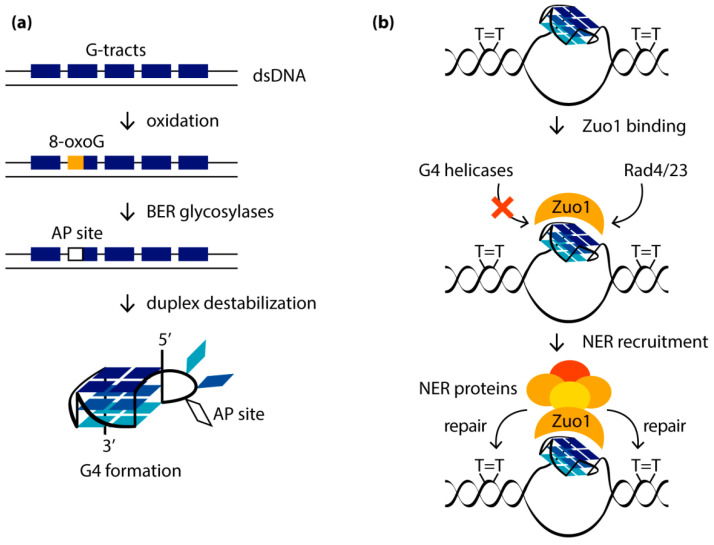
(**a**) Induction of BER-dependent G4 formation by oxidative stress. Removal of 8-oxoG by OGG1 glycosylase results in an AP site that destabilizes the DNA duplex and makes the formation of G4 by a G4 motif with five G-tracts more favorable. (**b**) Recognition, stabilization and protection against G4-unwinding helicases of G4 structures by the Zuo1 protein stimulates the repair of UV-induced damage (T=T corresponds to thymine dimer) via the NER pathway by recruiting the Rad4/23 complex.

The putative mechanism by which 8-oxoG activates G4 formation in the *VEGF* promoter and regulates gene transcription includes, first, the removal of the 8-oxoG from the double helix by the OGG1 protein to form an abasic site; the latter destabilizes the DNA duplex structure, allowing G4 to fold (Figure 6a) [131]. APE1 then binds to the abasic site extruded from the G4 by alternative quadruplex folding with participation of the spare intact G-tract. Binding of APE1 to DNA facilitates the interaction of the enzyme with transcription factors such as HIF-1α and AP-1, which induce gene expression.

A similar effect of oxidative base damage, 8-oxoG or abasic sites, on the BER-mediated regulation of transcription has been shown for the *PCNA* gene promoter, which has five G-tracts forming a parallel G4 [132]. Another study also confirmed that guanine oxidation in the G4 region of the *KRAS* promoter may have the epigenetic potential to control gene expression [133]. The G4-forming *KRAS* promotor sequence contains more than three dG residues in some G-tracts, as well as extra G-tracts, which provides a high G4 polymorphism and the possibility of its folding into three alternative quadruplex structures when some guanine residues are damaged. In this regard, it is important that DNA glycosylase OGG1, which removes the 8-oxoG lesion from the *KRAS* oncogene promoter in the DNA duplex structure, unproductively binds to folded G4 with oxidative base damage. As a result, 8-oxoG in the *KRAS* promoter, which is more abundant in G4-prone than in non-G4-prone regions, enhances the recruitment of two nuclear factors essential for transcription: the MYC-associated protein with zinc finger domain (MAZ) and heterogeneous nuclear ribonucleoprotein A1 (hnRNP A1 or A1), which activates KRAS expression. Thus, the overall effects of oxidative base damage in the *KRAS* promoter on transcription induction are substantially the same as those observed for the *VEGF* promoter; however, the protein partners involved in these processes are quite different.

## 9. G4 Activates the Removal of UV-Induced Damage by Nucleotide Excision Repair

In addition to the BER-mediated repair of oxidative base damage associated with the formation of G4 structures, oxidative-stress-dependent G4 folding has been shown to affect the nucleotide excision repair (NER) pathway, inducing its activation [134]. DNA damage caused by reactive oxygen species, followed by the abasic site generation by BER proteins, makes G4 formation more energetically favorable than maintaining a competing DNA duplex structure (Figure 6a). It was found that, especially after UV damage accompanied by oxidative stress, when more G4s are produced, Zuo1, identified as a novel G4-binding protein in yeast, is recruited to newly formed G4, presumably located in the immediate vicinity to UV-induced lesions, such as pyrimidine dimers. Binding of Zuo1 results in the stabilization of the G4 structure and contributes to the genome stability due to the recruitment of NER machinery proteins, particularly the Rad4/23 complex (Rad4 is the human XPC protein homolog and Rad23 is the human RAD23B homolog), which leads to efficient DNA repair (Figure 6b). The need for Zuo1 to bind G4 to stimulate NER is supported by the revealed UV sensitivity and the slower growth of Zuo1-deficient cells. This phenotype is unambiguously associated with a decreased level of G4 structures, since the cell doubling time can be restored in the presence of PhenDC3, an established G4-stabilizing small-molecule ligand. Using ChIP-seq and qPCR methods, it was shown that PhenDC3 stimulates the binding of the Rad4/23 complex to G4-forming genome regions in Zuo1-deficient cells.

In addition to the canonical repair pathways, post-replicative repair proteins such as the translesion protein Rev1 and the Pol θ are also associated with G4 formation. In cells lacking both functional Zuo1 and the NER machinery, the resulting G4 structures were accessible to translesion synthesis, as indicated by binding of the Rev1 protein. According to recent findings [134], the Zuo1 protein modulates the G4 level, protects quadruplexes from unwinding by G4-specific helicases, maintains NER function and regulates the selection of an appropriate DNA repair pathway near G4 structures. 

In another study, it was shown using ChIP-seq that helicases XPB and XPD, involved in transcription regulation and the NER pathway, also modulate the cellular level of G4s, which may further emphasize the importance of G4 formation for NER function [135]. Biochemical analysis showed that XPD effectively unwinds the G4 structures, while XPB specifically binds them.

## 10. Impact of G4 DNA on the Mismatch Repair Pathway

Mismatch repair (MMR) is required for proper maintenance of the genome by protecting against noncanonical base pairs or mismatches and insertion/deletion loops due to DNA polymerase errors or during homologous recombination. At the same time, this system has also been reported to be a driver of certain mutations, including disease-related instability of trinucleotide repeats in human cells [136]. It is important to note that the basic features of MMR have been conserved throughout evolution from bacteria to humans [137]. The most studied and widely employed MMR systems are those of *E. coli* and humans. In *E. coli*, the repair process is initiated by the binding of the MutS protein to mismatched bases. Upon recognition of the mismatch, MutS recruits MutL in an ATP-dependent manner to form a ternary complex that is believed to coordinate a cascade of subsequent events. MutL stimulates the MutH endonuclease, which interprets the absence of DNA methylation as a daughter-strand mark, thereby helping to distinguish and cleave the newly synthesized strand (methyl-directed MMR). In eukaryotes and most bacteria, MutL rather than MutH has the endonuclease activity (methyl-independent MMR). The unmethylated DNA strand is then hydrolyzed by a set of exonucleases. Finally, DNA polymerase and ligase fill the gap in the daughter strand (for review, see [138]).

The binding of *E. coli* MutS (ecMutS) protein and the human homolog MutSα to tetrameric polymorphic DNA G4s has been reported [35,139,140], and the affinity of these proteins for G4 turned out to be 2–4-times higher than for DNA with a G/T mismatch, a specific substrate of the MutS. The binding of MutSα to G4 motifs of immunoglobulin class-switching regions was directly visualized by electron microscopy [140]. The interaction of MutS with G4 has also been shown in vivo [139]. The analysis of the mode of MutS binding to G4s has generated great interest, since some studies have revealed variations in the ways ecMutS and its human homolog interact with quadruplex structures, on the one hand, and DNA mismatches, on the other [139,140]. Indeed, a highly conserved phenylalanine residue in the MutS’s Phe-X-Glu structural motif, critical for G/T mismatch recognition due to stacking with one of the mispaired bases, is not required for G4 recognition; in addition, the ATP-induced conformational changes in MutS, which promote the release of the mismatch-containing DNA duplex, are contrasted with the ATP-independent binding of MutS to the G4 structure. Ehrat et al. hypothesized that MutS is unable to activate the ATP-dependent canonical MMR pathway through G4 binding and that the function of MutS in G4 DNA metabolism is not associated with methyl-directed MMR [139]. However, no direct evidence has been obtained to support this hypothesis. To answer the questions of whether G4 formation interferes with mismatch-induced DNA cleavage caused by the coordinated actions of MutS, MutL and MutH proteins, and whether G4 itself activates MMR responses, our lab has designed new DNA constructs containing a biologically relevant intramolecular parallel G4 stabilized in the context of double-stranded DNA with a set of DNA sites required to initiate the MMR pathway (G/T mismatch and MutH recognition site). These DNA models were created by the hybridization of partly complementary strands, one of which contained a G4 motif d(GGGT)_4_ flanked by oligonucleotides, while the opposite strand lacked the site complementary to the G4-forming insert [35]. Using NMR spectroscopy, chemical probing, fluorescent indicators, circular dichroism and UV spectroscopy, the coexistence of parallel-stranded intramolecular G4 and duplex domains in the developed DNA models has been unequivocally proved.

In contrast to previous studies that used simple models with isolated G4, our DNA constructs allowed us, in addition to ecMutS, to study the affinity of G4 binding to other proteins involved in the initial steps of MMR: proteins MutL from *E. coli* (methyl-directed MMR) and MutS from *Rhodobacter sphaeroides* (methyl-independent MMR). Moreover, we were the first to assess the impact of the G4 structure on the functioning of ecMMR. We have proven experimentally that G4 is not perceived by ecMMR as damage that needs to be repaired; at the same time, this noncanonical DNA structure does not prevent the mismatch-dependent activation of MMR when the G4 and a G/T mismatch together are present in the DNA substrate at a distance of at least 17 bp.

**Figure 7:**
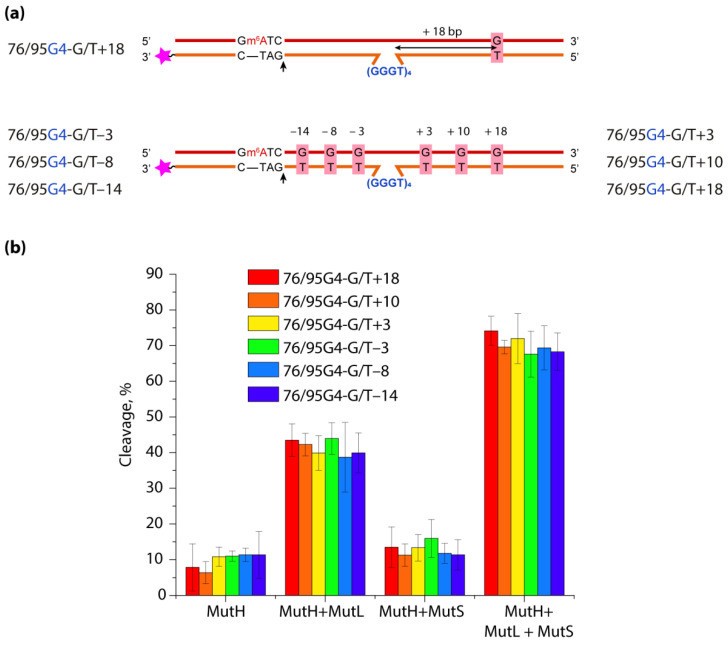
Efficiency of ecMMR protein-induced hydrolysis of linear DNA duplexes with an embedded parallel G4 and a MutH recognition site, which differ in the mismatch position. (**a**) DNA models used in this work; their names are shown on the left and right, and the sequences are the same as in [35]. 5′-Gm^6^ATC-3′/3′-CTAG-5′ corresponds to MutH recognition site, and black arrows indicate the position of DNA cleavage by the MutH endonuclease. Pink asterisks represent the TAMRA fluorophore at the 3′-end of the unmethylated daughter strand. 3′-Labeled cleavage products were separated from intact DNA strands by gel electrophoresis under denaturing conditions (7 M urea), allowing for evaluation of nicking potency; for the experimental conditions, see [35]. (**b**) Efficiency of DNA hydrolysis induced by ecMMR proteins (*p* < 0.05). Data were obtained for the MutH alone (250 nM concentration) and for the combinations of MutH with 250 nM MutS or 250 nM MutL, as well as with both 250 nM ecMutS and 250 nM ecMutL (all protein concentrations were calculated per monomer). The reaction mixtures were incubated at 37 °C for 1 h. These results have not yet been published.

To assess the role of the distance between G4 and DNA mismatch on the functioning of ecMMR on G4-containing substrates, a set of DNA duplexes with an embedded intramolecular parallel G4 structure and a monomethylated recognition site for the MutH endonuclease was prepared. They differed in the mismatch position—on the 3′- or 5′-side from G4—as well as the distance between the G/T pair and the G4 structure, which varied from 18 to 3 bp (Figure 7).

The efficiency of introducing a single-strand break into the prepared DNA models by ecMutH endonuclease alone and in the presence of ecMutL, ecMutS or both was evaluated and compared with the efficiency of control DNA duplexes lacking the G4 structure or G/T mismatch. It was shown that ecMutH alone cleaved all studied DNA substrates with equally low efficiency, while ecMutS added to the reaction slightly increased MutH activity, and the presence of MutL instead of MutS led to a ~4-fold growth in DNA cleavage efficiency (~40%) compared to MutH alone (~10%). Because ecMutH is known to colocalize and operate in coordination with ecMutL [141], the observed effect can be explained by the recruitment of an increased number of MutL molecules to G4, causing the activation of additional MutH molecules. Finally, the combined action of the full set of MMR proteins involved in the initial stage of mismatch repair—MutH, MutS and MutL—yielded the highest efficiency of MutH-mediated DNA cleavage (~70%) for all G4-containing DNAs, which was practically independent of the G/T mismatch position.

Thus, biologically relevant parallel G4 stabilized in double-stranded DNA does not trigger the initiation of ecMMR. At the same time, such a noncanonical structure as G4, capable of disrupting the movement of many processive proteins along B-DNA and binding with high affinity to the MutS and MutL proteins, does not block the action of endonuclease ecMutH, even when located in the immediate vicinity of a G/T mismatch and a MutH recognition site.

For the most studied MutS homologs, it was found that the mode of MutS binding to intermolecular and intramolecular G4s is apparently common for prokaryotic and eukaryotic organisms, regardless of the strand discrimination mechanism [35,139]. Their involvement in DNA repair and recombination is also well known [142,143,144]. It is believed that the interaction of MutS with G4 may play a yet unidentified role in the regulation of DNA recombination and G4 unwinding. Human MutSα and MutLα proteins have been shown to interact directly with the G4-resolving BLM helicase, although they do not affect helicase activity in vitro [145]. In addition, MutSα is able to inhibit the unwinding of the G4 DNA, but not the DNA duplex, by the FANCJ helicase [105].

## 11. Conclusions

It is known that G4s, the most actively studied noncanonical structures of nucleic acids, are capable of fine-tuning key biological processes. This review summarizes recent findings suggesting that G4 structures, in cooperation with various specialized proteins, lead to genetic instability through replication-dependent and/or replication-independent mechanisms, alter DNA availability to damaging agents and affect the efficiency and accuracy of DNA repair pathways.

Importantly, G4s can stimulate genetic instability both in the absence and presence of DNA damage. The main factors that play a dominant role in the efficiency of G4 damage have been characterized—mainly the introduction of oxidative guanine lesions, as well as their removal from the G4 structures by canonical repair pathways. These data support the conclusion that it is the special positions of the lesion in the G4 DNA, and not its general topology, that determines its repair efficiency; resistant lesions can be removed from the G4 through alternative folding mechanisms, making the lesion more accessible.

Of primary interest is the G4’s impact on the functioning of various repair pathways. While these noncanonical DNA forms stimulate the NER-mediated repair of UV-induced lesions, such as pyrimidine dimers, they demonstrate directly opposite effects on the BER machinery, suppressing the removal of oxidative base lesions from G4 motifs. Moreover, the preferential binding of the major MMR proteins, MutS and MutL, to G4 structures does not correlate with DNA mismatch repair activity, indicating an unexpected role of these DNA–protein interactions in genome maintenance.

The most important factors that modulate G4 stability and folding in the genome context and in vitro have also been summarized in this review.
